# *Staphylococcus aureus* Infections and Human Intestinal Microbiota

**DOI:** 10.3390/pathogens13040276

**Published:** 2024-03-24

**Authors:** Aotong Liu, Shari Garrett, Wanqing Hong, Jilei Zhang

**Affiliations:** 1 Department of Pharmacology & Regenerative Medicine, College of Medicine, University of Illinois Chicago, Chicago, IL 60612, USA; aliu52@uic.edu; 2 Division of Gastroenterology and Hepatology, Department of Medicine, University of Illinois Chicago, Chicago, IL 60612, USA; sgarre5@uic.edu; 3 Department of Microbiology and Immunology, University of Illinois Chicago, Chicago, IL 60612, USA; 4 Faculty of Science, University of Waterloo, Waterloo, ON N2L 3G1, Canada; w7hong@uwaterloo.ca; 5 School of Chemistry & Chemical Engineering and Materials Sciences, Shandong Normal University, Jinan 250061, China

**Keywords:** *Staphylococcus aureus*, bacterial translocation, gut microbiome, microbial homeostasis, dysbiosis

## Abstract

*Staphylococcus aureus* (*S. aureus*) is a common pathogen that can cause many human diseases, such as skin infection, food poisoning, endocarditis, and sepsis. These diseases can be minor infections or life-threatening, requiring complex medical management resulting in substantial healthcare costs. Meanwhile, as the critically ignored “organ,” the intestinal microbiome greatly impacts physiological health, not only in gastrointestinal diseases but also in disorders beyond the gut. However, the correlation between *S. aureus* infection and intestinal microbial homeostasis is largely unknown. Here, we summarized the recent progress in understanding *S. aureus* infections and their interactions with the microbiome in the intestine. These summarizations will help us understand the mechanisms behind these infections and crosstalk and the challenges we are facing now, which could contribute to preventing *S. aureus* infections, effective treatment investigation, and vaccine development.

## 1. Introduction

*Staphylococcus aureus* is a microbe carried by humans. It can cause minor, severe, or fatal infections under particular circumstances, such as minor skin and soft-tissue infections, food poisoning, and life-threatening diseases like bacteremia and infective endocarditis [1]. Moreover, it is one of the five most common causes of hospital-acquired infections and one of the primary causes of wound infections following endocarditis surgery [1,2]. *S. aureus* infections trigger the immune response, both innate and cell-mediated, activating numerous immune cells, such as monocytes, dendritic cells, and macrophages, and signaling pathways, such as Toll-like receptors (TLR-1, TLR-2, and TLR-6), interleukins (IL-4, IL-6, IL-10, and IL-13), chemokines (CXC ligand-1 and CXC ligand-2), and T-helper 1/2 [3]. Meanwhile, *S. aureus* colonizes around 20-30% of the human population persistently in the nose and frequently in other body sites like skin, throat, axillae, groin, and intestine [4,5]. As we delve deeper into understanding the intricate interplay between potential pathogens and the host microbiota, the significance of various commensal microbiota in influencing *S. aureus* colonization is becoming increasingly apparent. [5,6]. 

Our body harbors more microbial cells than human cells. They constitute our microbiota system, and their encoded genes are the microbiome. This complex ecological community is considered our “forgotten organ” nowadays. The microbiota in this system is most heavily colonized in the gastrointestinal tract and skin. It can significantly impact our health and physiology by interacting with each other and the host. The intestinal microbiome plays a critical role in numerous biological functions and activities, not only in digestion-related energy harvesting and storage and metabolic processes such as fermenting and absorbing undigested carbohydrates but also in modulating infections and activating the immune system [7,8]. With the increasing number of related studies, we will continue to learn more about the gut microbiome’s influence on other organs and tissues’ physiological functions and immunity far beyond the intestine [8,9,10,11]. 

The balance of the intestinal ecosystem is closely related to human and animal health. It has been widely reported that interactions among the intestinal microbiota are involved in the pathogenesis of inflammatory bowel diseases and other infections [12,13,14]. For instance, an oral infection of *S. aureus* could destroy the mouse-stable intestinal microbiota structure. However, these microbial alterations can be improved by treating with lactic acid bacteria, which play an essential role in maintaining the integrity of the gut mucosal barrier [15,16]. The impacts of the intestinal microbiota on *S. aureus* infections are also found in the other organs but with different influences and mechanisms, such as resistance to *S. aureus* pneumonia through Type 17 (Th17) cells in the gastrointestinal tract coated with segmented filamentous bacteria (sfb), and its contribution to the development of *S. aureus*-induced mastitis in mice by disrupting gut microbiota homeostasis, characterized by an increased abundance of pathogenic *Enterobacter* bacteria and a reduced abundance of short-chain fatty acid (SCFA)-producing bacterial phyla [17,18]. Gut microbial dysbiosis could cause dysfunctions of the intestinal mucosa barrier and other blood–tissue barriers, such as the blood-brain barrier (BBB) and blood-testis barrier (BTB). Their functional maintenance needs the gut microbiota, which could be influenced by the increased BBB and BTB permeability in germ-free mice [19,20]. Even though colonization of *S. aureus* in healthy adults did not significantly affect gut microbiota diversity, the alteration of the gut microbial composition and microenvironment could imbalance the mucosal barrier and local immune ability and then facilitate *S. aureus* colonization, leading to further dysfunction in the immune system and blood–tissue barriers in other locations of the body. 

In this review, we summarized recent progress in understanding the interactions between *S. aureus* infections and the microbiome in the intestine. These summarizations will help us understand the mechanisms behind these interactions and the challenges we are facing now, which could contribute to preventing *S. aureus* infections, investigating effective treatment, and developing vaccines.

## 2. *Staphylococcus aureus*-caused Skin and Soft-Tissue Infections (SSTIs)

*Staphylococcus aureus* is a Gram-positive cocci-shaped species of bacteria in the Staphylococcaceae family. It is commonly found in the environment and the normal human microbial system, primarily on healthy skin and mucous membranes [21]. About 30% of humans carry *S. aureus* in their noses, which is mostly safe. Still, it can cause complications and severe infections in local organs and intestines, such as *S. aureus* infections on the skin and soft tissues, by entering the bloodstream and traveling to internal tissues [22]. 

Although *S. aureus* usually acts as part of the commensal microbiota, the emergence of antibiotic-resistant strains such as methicillin-resistant *Staphylococcus aureus* (MRSA) has become a global problem in clinical medicine. MRSA is a strain of *S. aureus* that has developed or acquired resistance to β-lactam antibiotics [23]. MRSA strains carry an antibiotic resistance gene, *mecA*, which allows them to stop β-lactam antibiotics from inactivating enzymes critical for cell wall synthesis [24]. MRSA has become the leading cause of skin and soft-tissue infections (SSTIs) in recent decades due to its emergence as a community-associated methicillin-resistant *Staphylococcus aureus* (CA-MRSA). However, it originally began as a hospital-acquired infection [1,25,26,27,28,29]. Recently, reports indicated that emergency department (ED) visits for SSTIs in the United States increased from 1.2 million in 1993 to 3.4 million in 2005 [30,31,32,33]. 

*S. aureus* SSTIs can range from benign, such as impetigo and uncomplicated cellulitis, to immediate and life-threatening. *S. aureus* is the most common pathogen isolated from surgical-site infections (SSIs), cutaneous abscesses, and purulent cellulitis [1]. Impetigo is the most common bacterial skin infection in children. It generally presents as bullous or popular abrasions on the face or extremities that progress to crusted lesions without any accompanying systemic symptoms [34]. Even though *S. aureus* purportedly causes a minority of non-purulent cellulitis cases, the true etiology of this condition is still unclear due to the lack of diagnostic gold standards and the variability of the methods introduced by different microbiological groups [35]. Moreover, SSIs occur in 2% to 5% of patients after surgery if the heterogeneity of the type of procedure, population studied, comorbid illness, surgeon experience, setting, and antimicrobial prophylaxis utilization were considered in the diagnosis [36]. The pathogenesis of *S. aureus* infections involves the whole immune system, in which neutrophil response is the primary defense against the bacteria by migrating to the infection site where *S. aureus* enters the skin. However, the pathogen could evade the immune response in several ways, such as blocking leukocytic chemotaxis, sequestering host antibodies, hiding from detection via polysaccharide capsules, or biofilm formation [1]. 

**Figure 1:**
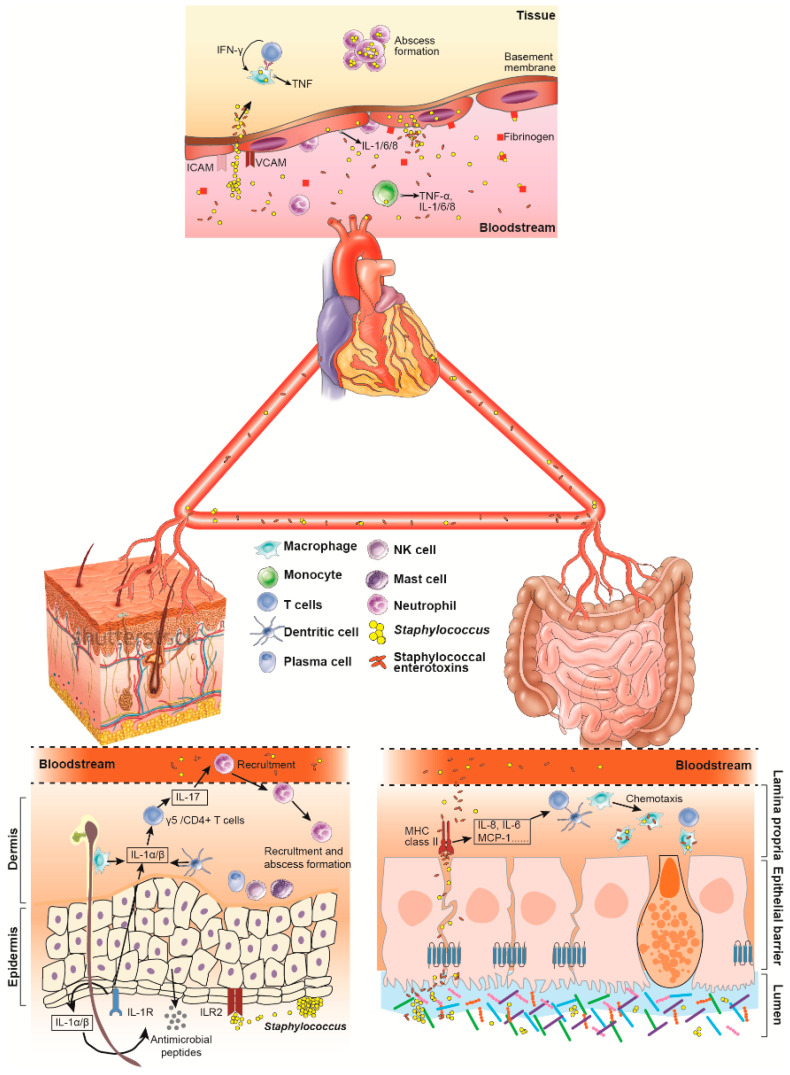
*Staphylococcus aureus* infections in organs/tissues. In the heart, the first response to *S. aureus* infections is the development of proinflammatory cells, including neutrophils, monocytes, and macrophages, which is supported by the production of cytokines (TNF-α, IL-1, IL6, and IL-8), adhesion molecules (ICAM, VCAM), integrins, and tissue factors. The cytotoxins released by *S. aureus* could trigger the immune response, both innate and cell-mediated, of T-cells and B-cells. During skin infections, immune cells, including dendritic cells, macrophages, mast cells, B- and T-cells, plasma cells, and natural killer (NK) cells, produce proinflammatory cytokines, chemokines, and adhesion molecules. These cytokines could induce the production of antimicrobial peptides that exhibit bacteriostatic or bactericidal activity against *S. aureus*. Lipoproteins and lipoteichoic acid of *S. aureus* activate Toll-like receptor 2 (TLR-2), while IL-1α and IL-1β activate interleukin-1 receptor (IL-1R), which promotes the production of IL-17 and cytokines from T-cells. In the intestine, the inflammatory injury caused by staphylococcal enterotoxins is mediated by MHC class II-expressing mucosal professionals (macrophages and dendritic cells) and non-professional (like myofibroblasts) CD4+ T-cells. These related processes can produce solid proinflammatory cytokines and chemokines, such as IL-6, IL-8, and MCP-1. *S. aureus* or its enterotoxins could cross the local epithelial barrier and reach the bloodstream, and then further translocate to other organs/tissues and cause bacteremia, or vice versa.

SSTI-induced immune responses are meticulously coordinated defense mechanisms engaged by innate and adaptive immune systems at various infection stages. Upon entry through exposed epithelial surfaces such as the skin, *S. aureus* engages with epithelial cells through a process primarily orchestrated by the Toll-like receptor (TLR)-2-mediated local production of soluble mediators, including cytokines, chemokines, and antimicrobial peptides. Following cell and tissue invasion, the transition to an inflammatory phase occurs, where intracellular microbial sensors and inflammasomes are activated for bacterial clearance. Concurrently, tissue-resident mast cells and macrophages work to recruit a suite of immune cells, including neutrophils, macrophages, and NK cells, fostering an inflammatory response that supports the generation of IL-17-producing NKT, γδ T-cells, and T-helper cells [37]. Antimicrobial peptides and pattern recognition receptors against SSTIs further bolster this innate immune response. Among these initial responses, the keratinocytes and neutrophils play a crucial role, while dendritic cells and T-lymphocytes provide a supporting role later in the infection [37]. Meanwhile, interleukin-17-producing T-cells emerge as pivotal players in the adaptive immune response to the *S. aureus* SSTIs, which has the potential therapeutic benefits in targeting these T-cells in SSTI patients [38,39] (Figure 1). On the other hand, *S. aureus* has crafted immune evasive strategies on the bacterial front, enabling them to elude protective immune responses in the host. These strategies encompass the secretion of specific products like staphylococcal protein A (SpA), staphylococcal binder of immunoglobulin (Sbi), and adenosine synthase A (AdsA), all aimed at thwarting the host’s immune system [40]. Moreover, *S. aureus* isolated from SSTIs was characterized by substantial molecular heterogeneity along with a high prevalence of Panton–Valentine leucocidin (PVL) genes, underscoring a genetic underpinning for the pathogenicity and immune interaction of the bacteria and host [41]. 

The skin and intestine maintain homeostasis and fight against pathogenic microbes by relying on multifaceted mechanisms. The skin is an active immune organ whose function is augmented by commensal microbiota on its physical barrier, formed by numerous layers of epidermal and dermal keratinocytes [42]. Like the skin, the intestine is filled with a distinct microbiota often recognized as an “ignored organ.” It is critical in maintaining health and defending against disease [8], including infections caused by *S. aureus*. To sustain host health, the skin and intestine must maintain homeostasis with the abundance and diversity of commensal organisms on their epithelial surface. The abundance and diversity of the microbiota in the skin and intestine can impact and shape host–microbe interactions, especially in neonatal and early life [43,44]. On the one hand, *S. aureus* infection in the skin could destroy microbial homeostasis by inhibiting colonization and contributing to dysbiosis [45]. Enriched *S. aureus* in the skin could further impact the abundance and diversity of the microbiota in the gastrointestinal tract through microbial translocation via the oral–fecal route and/or by microbial products in the bloodstream. Conversely, the cutaneous immune response to *S. aureus* involves innate and adaptive immune systems [38]. Activation of the immune system impacts intestinal immunity, which could have further implications on the microbial balance in the gut. Gut dysbiosis could significantly affect skin homeostasis, influencing the pathogenesis, symptoms, and prognosis of *S. aureus* infections. As one of the most common bacteria that causes breast infections and mastitis, *S. aureus*, colonized on the skin, can enter the body through a break in the skin, usually on the nipple [46]. These bacteria could relocate to the digestive system through the bloodstream and further impact intestinal microbial homeostasis. These interactions between the gut microbiota and *S. aureus*-caused mastitis were investigated with a mouse model. Increased blood–milk barrier permeability and mastitis severity were observed in mice with gut microbial dysbiosis compared to controls, and these effects could be reversed by feces microbiota transplantation (FMT). These effects were most likely regulated through short-chain fatty acids (SCFAs) for the administration of sodium propionate and sodium butyrate, and probiotics (butyrate-producing bacteria) could reverse the changes in the blood–milk barrier and reduce the severity of mastitis induced by *S. aureus* [18]. More specifically, cholic acid (CA) and deoxycholic acid (DCA) were involved in this gut microbiota-mediated process. These secondary bile acids alleviate *S. aureus*-induced mastitis through TGR5-cAMP-PKA-NF-κB/NLRP3 pathways in mice [47]. However, additional studies and research must be performed to investigate the mechanisms behind these hypotheses. 

## 3. *Staphylococcus aureus* Caused Food Poisoning

Staphylococcal food poisoning (SFP) is widespread and is becoming one of the most common causes of foodborne disease, with the case number continuously increasing since it was first reported [48,49,50]. *Staphylococcus* spp. exotoxins, the primary food-poisoning agents, are a group of low-molecular-weight pyrogenic proteins grouped into three families depending on their amino acid sequence. These families include Staphylococcal enterotoxins (SEs), Staphylococcal enterotoxin-like (SEls), and the toxic shock syndrome toxin 1 (TSST-1) [48]. As a superfamily, the SEs have structural and functional similarities and possess potent super-antigenic activity, causing disruptions in adaptive immunity. They can be further separated into classical (SEA-SEE) and newer (SEG-SE/γ and counting) enterotoxin groups [50]. However, the categories of enterotoxins are complicated. By comparing their nucleotide and amino acid sequences using newly developed sequence technologies, the 24 currently identified SEs and SEls can be further divided into evolutionary groups: the SEA group with SEA, SED, SEE, SE/J, SHE, SEN, SEO, SEP and SES; the SEB group with SEB, SECs, SEG, SER, SE/U and SE/W; the SEI group with SEI, SEK, SEL, SEQ, SEM, and SE/V; and the SE/X group with TSST-1, SET, SE/X, SE/Y and members of another group of SEs called super-antigen-like toxins [50,51]. Therefore, it is not surprising how complex the network of regulatory pathways used by *S. aureus* is in controlling these toxin productions. It was suggested that a combination of quorum-sensing (QS) and other two-component systems (TCSs) and many trans-acting regulatory proteins is used by *S. aureus* to quickly make changes in the regulation of genes associated with important physiological features, including drug resistance metabolism, immune evasion, and virulence [52,53,54]. The accessory gene regulator (Agr) QS system is activated at high cell densities and comprises two transcriptional units transcribed in opposing directions: RNA-II codes for four genes—*agrA*, *agrB*, *agrC*, and *agrD*—and regulatory RNA RNA-III [50,55]. 

Meanwhile, SFP complexity is underscored due to the molecular heterogeneity observed in SFP outbreaks. In a reported and documented foodborne outbreak, a spectrum of enterotoxin genes, including *seg*, *sei*, *sem*, *sen*, *seo*, and *selu*, were identified in *S*. *aureus* isolated from both the contaminated food and affected patients, although there was an absence of classical SE production [56]. This variability of enterotoxin genes hints at a broader spectrum of immune interactions and responses in SFP outbreaks, which emphasizes the exigency for a deeper understanding of the molecular and immunological landscapes of SFP. Recently, the report of *S. aureus* as one of the commonest agents of foodborne diseases due to its enterotoxins highlights the importance of the molecular epidemiological characterization of *S. aureus* in understanding the dynamics and spread of SFP [57]. Moreover, the heat-stable nature of these enterotoxins, particularly SEs, makes them resilient to food processing and cooking temperatures, posing a significant challenge in controlling SFP [58].

In addition to coagulase-negative strains, which are considered non-pathogenic, different Staphylococcal species can produce an array of exotoxins that trigger diverse response pathways in infection. For SEs, picomolar concentrations can cause toxic shock syndrome, fever, hypotension, and multi-organ failure [59,60]. These toxins cause emesis and are resistant to heat, acidity, and hydrolysis, mediated by most proteolytic enzymes. In addition, SEs can cause the aberrant activation of the immune system by non-specifically interacting with T-cells and macrophages, resulting in T-cell activation and increased cytokine release [48,61,62]. Meanwhile, SEIs are capable of general immunomodulation and can induce neither emesis nor T-cell activation [60,63]. TSST-1, similar to SEs, can directly activate macrophages and T-cells [60]. 

Unlike conventional antigens, SEs bridge antigen-presenting cells (APCs) and T-cells by activating the T-cells independently from antigen processing and presentation to the T-cells by APCs [50]. Typically, SEs first bind to the major histocompatibility complex (MHC) class II molecules located on APCs and one or more variable beta chains (Vβ) of T-cell receptors (TCRs) coordinately [64,65]. However, other receptors have been described as involved in this process, such as the variable alpha (Vα) chain targeted by SHE [66]. Moreover, the maximal super-antigenic activity of SEB needs additional co-stimulatory receptors on T-cells (CD28) and APCs (B7-2) [67,68]. SE activity is characterized by polyclonal activation of a large pool of CD4+ and CD8+ cells regardless of the mechanism of cross-linking, followed by a massive release of an assortment of T-helper 1 (Th1) cytokines, like tumor necrosis factor (TNF) α, interleukin 1 (IL-1), IL-2, and interferon (IFN) γ [69,70,71,72]. Moreover, a list of immune cell types has been recognized to be targeted by the SEs directly or indirectly, including neutrophils, γδ T-cells, invariant natural killer T (iNKT)-cells, B-cells, mast cells, and mucosa-associated invariant T (MAIT)-cells [73,74,75,76,77,78] (Figure 1). 

Staphylococcal exotoxins have huge influences on the digestive tract. Staphylococcal exotoxins can cause cytotoxicity in intestinal cells, resulting in gastroenteritis, vomiting, and gastric inflammation [61]. Reports indicate that SEs have an affinity for intestinal epithelial cells and mucus-producing goblet cells, which can be used as gateways to traffic across the intestinal epithelia to reach their final targets in other body parts [79,80,81]. These findings were also confirmed with mouse models, in which *S. aureus* infection induced damage in the small intestine and disrupted host structures essential for epithelial integrity [82]. The intestinal epithelial cell barrier is the first line of defense against pathogenic organisms and is critical for maintaining microbial homeostasis. Goblet cells are the primary producers of mucins, and their secreted proteins protect the intestinal epithelial lining [83]. Notably, it is thought that the movement of these enterotoxins through gut epithelial cells was a glycolipid-dependent transcytosis process that may be facilitated in the presence of other staphylococcal virulence determinants [84]. These goblet and intestinal epithelial cells are often targeted by SEs, suggesting that *S. aureus* infections may cause permanent damage to the intestinal epithelium and alter the microbiota, which could increase the host's sensitivity to SEs in the future. The chronic microbial imbalance caused by *S. aureus* could further impact physiological health and organs beyond the gut. More studies are needed to determine the potential future impacts of these influences. 

The crosstalk between *S. aureus* and host intestinal cells, especially intestinal epithelial cells, has been reported. Not surprisingly, *S. aureus* infections can alter the overall structure of the intestine microbiota and the microbial metabolic profiles by damaging the intestinal epitheliums and/or through the bacterial products. For example, in *S. aureus*-infected colonic Caco-2 cells, a significant decrease in the localization and specific hydrolytic activities of SI (sucrase–isomaltase) toward sucrose and isomaltose (palatinose) was observed in the BBMs (brush border membranes) (P2 fraction) 48 hours post-infection. The specific SI activities increased in the basolateral membrane/intracellular fraction (P1) [85]. Moreover, it seems like *S. aureus* can compromise colonization resistance by the colonic microbiota because a number of *S. aureus* cells stabilized until they were washed out, while populations of indigenous bacteria were transiently altered [86]. Luckily, these alterations of microbial dysbiosis and the colonization of *S. aureus* could be reversed and blocked by treating them with probiotics or their products. For instance, the *B. subtilis* probiotic could eliminate more than 95% of the total *S. aureus* colonized in the human body [87]. The mixed lactic acid bacteria could retain an intestinal microbiota composition similar to the control group with the most abundant taxa of *Bacteroidales*, *Lachnospiraceae*, *Bacteroides*, and *Prevotellaceae* [16]. One potential pathway could be regulating intestinal barrier function through CYP1A1 (cytochrome P4501A1). It was reported that mice with a dysfunction of this pathway showed an altered gut microbiome, a reduced metabolic shift from lysine to cadaverine, antimicrobial molecular production (Retnlb, Gbp7, and Gbp3), and protection against gut barrier disruption from MRSA challenge [88]. 

## 4. *Staphylococcus aureus* Caused Bacteremia

Bacteremia is perhaps the best-described manifestation of *S. aureus* infection to date. *Staphylococcus aureus* bacteremia (SAB), caused by *S. aureus,* the second-most isolated pathogen in the hospital inpatient setting, requires complex medical management, resulting in substantial healthcare costs [89,90]. Most nosocomial and community-acquired SAB is secondary to MRSA and is associated with a high mortality rate and increased hospital stay [91]. In the USA, *S. aureus* caused 13.2% of nosocomial bacteremia between 2011 and 2014 [89,90]. It also affects 10 to 30 people per 100,000 annually in the industrialized world [92]. 

Although the mechanisms of SAB are largely unknown, the high-risk factors for SAB development are age (infants and seniors), additional comorbidities (diabetes, renal disease, AIDS), the presence of in-dwelling medical devices, intravenous drug use, and low socioeconomic status [93]. Patients with dermal *S. aureus* infection can develop SAB. The dermal mode of infection is a standard entry mechanism for *S. aureus* to infect deeper tissues and the bloodstream [94,95]. SAB can lead to sepsis, a paradoxical immunosuppressive response that is occasionally concurrent with inflammation. This general scheme of sepsis could be caused by the multiple virulence mechanisms of *S. aureus*, including directly targeting specific immune responses or utilizing the host’s coagulation system to bind to the endothelium [96]. Moreover, reports indicate that SAB can also cause endocarditis [96]. 

The pathogenesis of SAB is a complex interplay between the pathogen and the immune system of its host. *S. aureus* can enter the bloodstream and evade the host’s immune responses through its arsenal of virulence factors, such as various toxins and super-antigens [97]. These virulence factors can directly target immune cells or exploit host immune systems, which could cause a systemic inflammatory response that leads to severe conditions like sepsis or endocarditis, as we mentioned above [97]. Among critically ill patients, sepsis is a leading cause of death in the early disease course as a result of a dysregulated proinflammatory phase (systemic inflammatory response syndrome [SIRS]) characterized by high levels of IL-1β, TNF-α, and monocytes expressed with human leukocyte antigen (HLA)-DR, or of a later predominating immunosuppressive recovery phase (compensatory anti-inflammatory response syndrome [CARS]) characterized by anti-inflammatory IL-10 and monocytes with reduced expression of HLA-DR [98,99]. Moreover, T-helper cells, such as Th1 and Th17, have been reported to be critical in patients infected with *S. aureus* [100,101] (Figure 1). 

The gastrointestinal tract may be another location for *S. aureus* to enter the bloodstream and disturb gut microbial homeostasis. Immunodeficiency caused by other risk factors, such as inflammatory bowel disease (IBD), diabetes, HIV infections, and food poisoning of *S. aureus*, can impair the intestinal protective barrier, allowing pathogens to permeate the gut epithelium and enter the bloodstream. Reversely, when patients develop SAB, which leads to sepsis and damage to the immune system, it can impact intestinal function and microbial balance in the gut lumen. These imbalances can be supported by the adherence of *S. aureus* to human intestinal mucus [102] and alterations of the microbial diversity in the gut caused by the colonization of *S. aureus* [103]. This also suggested that the integrity of the gut epithelial layer, rather than the pathogenic potential of the investigated enteric *S. aureus* isolates compared to the bloodstream, determines whether staphylococci from the gut microbiome will become invasive pathogens [104]. The rise and persistence of mutations altering virulence and antibiotic susceptibility during *S. aureus* bacteremia, intestinal dissemination, and transmission were linked to the production of virulence factors based on gene deletion studies of the *sae* and *agr* two-component systems [105]. 

## 5. *Staphylococcus aureus* Caused Endocarditis

Historically thought to be caused by *viridians streptococci*, *S. aureus* is the worldwide leading cause of infective endocarditis (IE) [1,93]. Moreover, *S. aureus* has become increasingly problematic for IE due to its propensity to cause severe diseases and frequent antibiotic resistance. The proportion of *S. aureus* prosthetic valve endocarditis (PVE), one of the most morbid bacterial infections, has grown in recent decades due to methicillin-resistant *S. aureus* (MRSA) [106]. Although the frequency is relatively low and related studies are limited, IE has been well studied by extensive multinational collaborations, such as the International Collaboration on Endocarditis Prospective Cohort Study (ICE-PCS). In addition, robust population-level studies have provided critical insights into the epidemiology and prognosis of IE and *S. aureus*-induced IE [1,93,107,108]. Therefore, we now know that *S. aureus* endocarditis (SAE) is one of the most severe manifestations or complications of *S. aureus* infections and is associated with more severe IE than is demonstrated by other bacterial pathogens [93,109]. Although reports indicate lower fatality rates, SAE is generally associated with 20-30% in-hospital mortality [93,110]. 

Bacterial colonization and infection begin with damage to the cardiac endothelium by formatting a nidus. Then, the production of extracellular matrix proteins, tissue factors, and deposition of fibrin and platelets to form sterile vegetations was elicited by the exposed subendothelial cells [111]. Meanwhile, reports show that *S. aureus* has several cell wall-associated factors that allow it to attach to extracellular matrix proteins, fibrin, and platelets [1]. For instance, clumping factors A and B (ClfA and ClfB) are essential for the attachment to and colonization of the valvular tissue. At the same time, fibronectin-binding proteins A and B (FnBPA and FnBPB) could facilitate binding to fibrinogen and fibronectin and play a critical role in subsequent endothelial invasion and inflammation [112,113]. 

As a severe cardiac condition, IE is characterized by the formation of an infected blood clot or vegetation attached to a cardiac valve accompanied by inflammation and valve destruction [114]. These clots, formed by the interaction of *S. aureus* with platelets (platelet-fibrin clots), shield the bacteria from host immune cells and help them stay amply within heart tissue, leading to endocarditis [115]. One of the mechanisms is through surface proteins and adhesins. The combination of *S. aureus* protein A (SpA) and the Fc region of antibodies prevents the opsonization of *S. aureus*, thereby inhibiting immune cell phagocytosis [116]. Meanwhile, clumping factors (ClfA and ClfB) and fibronectin-binding protein A (FnbpA) play a pivotal role in the adherence of *S. aureus* to fibrinogen and platelets. This adherence promotes the bacterial colonization of heart valves and leads to bacterial vegetation in endocarditis [117,118]. Moreover, neutrophils, a type of white blood cell that serves as the body’s first line of defense against bacteria invasions, have been found to play an essential role in the context of IE. They detect and swiftly engulf *S. aureus* through neutralization with a lethal cocktail of antimicrobial enzymes and reactive oxygen species inside neutrophils and neutrophil extracellular trap (NET) web-like structures [114,118]. Meanwhile, the expression of immune evasion proteins, like chemotaxis inhibitory proteins of *S. aureus* (CHIPS) and staphylococcal complement inhibitor (SCIN), is the basement for other reported mechanisms, to avoid the attack of the complement system [119]. One of the critical aspects of IE is the variable and complex host immune alterations, which are strongly regulated by the bacterial cell itself, the bacterial virulence profile, and certain host circumstances of the host immune environment, the factors of which are largely unknown [120,121,122] (Figure 1).

Using next-generation sequencing, Di Bella et al. found bacterial DNA sequences in the aortic/mitral valves from patients having either evidence or history of infectious diseases, particularly infective endocarditis [123]. This finding highlights bacterial colonization in the cardiac valves. As a complex entity with rapid evolution in the nosocomial environment and a commensal bacterium inhabiting the human gastrointestinal tract, *Enterococcus faecalis* is the causative agent for EFIE (*Enterococcus faecalis* infective endocarditis) [124]. A report recently indicated the possible association between *Enterococcus faecalis* endocarditis and colorectal neoplasms [125]. *Enterococci* display an array of defensive strategies in addition to alterations in the gut microbiome that might lead to poor outcomes, like systemic immune changes and bacterial translocation from the gut to the heart. 

## 6. Intestinal Microbial Alterations in *S. aureus* Infections

We discussed above the importance of the gut microbiome in regulating health and diseases when it is in homeostasis status. This is mainly based on microbial diversity and abundance in the intestine. When pathogenic infections happen, including *S. aureus* infections, the microbial balance could be destroyed in both in situ infections and organs/tissues far away, such as the intestine. It has been demonstrated that microbial community changes in the feces of cows with mastitis caused by *S. aureus* were similar to those in milk, characterized by an increased abundance of *Enterococcus*, *Streptococcus*, and *Staphylococcus* and a decreased abundance of *Lactobacillus* [126]. Similarly, in a murine mastitis model induced by *S. aureus*, the abundance of pathogenic *Enterobacter* bacteria increased, while short-chain fatty acid (SCFA)-producing bacteria *Firmicutes* and *Bacteroidetes* were discovered to decrease [18]. Moreover, humans carrying methicillin-sensitive *S. aureus* (MSSA) exhibited a higher proportion of *Parasutterella* and *Klebsiella* but a lower abundance of *Bacteroides* [103]. Consistent with this, a significant, transient decrease in the numbers of *Bacteroides*, *Bifidobacterium*, and *Lactobacillus*/*Enterococcus* after inoculation of *S. aureus* was found in an in vitro colonic model system to mimic infection in vivo [86]. Moreover, butyric acid, one of the critical short-chain fatty acids (SCFAs), was significantly decreased due to *S. aureus* infection, which strongly supported the importance of intestinal SCFA-producing bacteria in *S. aureus* infection [86]. As the intestinal microbiota includes bacteria, viruses, fungi, archaea, and other organisms, more studies should be performed to study the impacts of *S. aureus* infections on other microorganisms in the gut.

## 7. Preventive and Treatable Strategies

**Figure 2:**
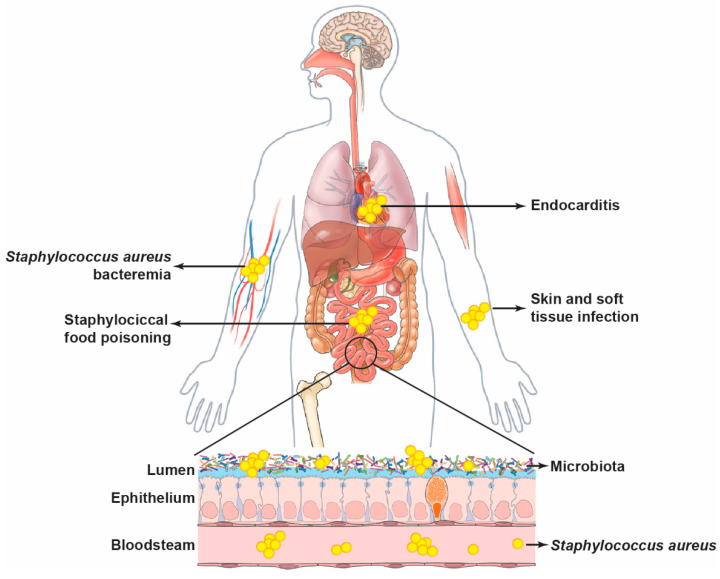
*Staphylococcus aureus* infection and gut microbial homeostasis. *S. aureus* can cause skin and soft-tissue infections, endocarditis, food poisoning, and bacteremia and influence microbial homeostasis by impacting the intestinal immune system or increasing bacterial translocation through the bloodstream. Reversely, gut microbial dysbiosis could affect *S. aureus* infection or sensitivity to the pathogen by microbial products or bacterial translocation through the bloodstream.

*S. aureus* infections such as SFP, SAB, and SAE are generally recognized as acute diseases. The acuteness of their conditions may be the reason for the limited studies on the interactions between the gastrointestinal microbiota and *S. aureus* infection (Figure 2). However, research demonstrates that *S. aureus* could adhere to human intestinal mucus [102], and its colonization could impact gut microbial ecology in both human and mouse models [16,86,103]. All these studies highlight the critical role of *S. aureus* in intestinal microbial homeostasis. The dysbiosis of *S. aureus* and other microbiota in the intestine could lead to functional issues in the intestinal immune system, which could further impact the whole immune status or bacterial translocation from the gut to other tissues or organs through the bloodstream. Therefore, an alternative and promising approach to therapeutic development should focus on maintaining gut microbial homeostasis through prebiotics, bacterial peptides, or fecal microbiota transplantation (FMT), further limiting susceptibility to *S. aureus* infection.

Short-chain fatty acids (SCFAs), mainly acetic acid, propionic acid, and butyric acid, are a group of bacterial metabolites produced by specific bacteria in the intestinal tract [127]. As discussed above, SCFAs play a critical role in *S. aureus* infections. Treatments of sodium propionate, sodium butyrate, and probiotics (butyrate-producing bacteria) could reverse the changes in the blood–milk barrier and reduce the severity of mastitis induced by *S. aureus* in a mouse model [18]. SCFAs have been found to have multiple effects on both the local intestine and more distal organs, e.g., the brain [127]. Using SCFAs as a potential treatment for or the prevention of *S. aureus* infections is more than possible.

The other potential therapeutic methods for *S. aureus* infections could be probiotics, which have been used to enhance body immunity, improve digestive system health, and prevent/treat multiple diseases. It has been reported that treatment with mixed lactic acid bacteria could prevent *S. aureus* infection in mice and improve the structure of the intestinal microbiota destroyed by *S. aureus* infection [16]. Encouragingly, a clinical trial on *S. aureus*-colonized participants demonstrated that *Bacillus subtilis* probiotics could eliminate more than 95% of the colonized *S. aureus* from the human body without altering the intestinal microbiota [87].

Fecal microbiota transplantation (FMT), a therapy being rapidly accepted that has gained increased interest recently, could also be used for *S. aureus* infections since it has been recommended for conditions like autoimmune disorders, certain allergic diseases, and metabolic disorders that are not intestinal diseases [128]. It has been found to mediate the protective role in endometritis and mastitis induced by *S. aureus* in mice [18,129]. It could also restore the dysbiosis caused by MRSA in patients with enterocolitis [130]. All these factors indicate the high possibility of FMT being used as a therapy for *S. aureus* infections.

## Abbreviations

SSTIs	*Staphylococcus aureus*-caused skin and soft-tissue infection
MRSA	Methicillin-resistant *Staphylococcus aureus*
CA-MRSA	Community-associated methicillin-resistant *Staphylococcus aureus*
SSIs	Surgical-site infections
SFP	Staphylococcal food poisoning
SE	Staphylococcal enterotoxins
SEI	Staphylococcal enterotoxin-like
SAB	*Staphylococcus aureus* bacteremia
IBD	Inflammatory bowel disease
IE	Infective endocarditis
SAE	*S. aureus* endocarditis
TNF	Tumor necrosis factor
IL	Interleukins
NK-cells	Natural killer cells
TLR	Toll-like receptor
MCP-1	Monocyte chemoattractant protein-1
VCAM	Vascular cell adhesion molecule
ICAM	Intercellular cell adhesion molecule
